# Supplementary material for: Evaluation of the Mammalian Aquaporin Inhibitors Auphen and Z433927330 in Treating Breast Cancer
Source: Cancers (Basel). 2024 Jul 30;16(15):2714. doi: 10.3390/cancers16152714 (PMC11311482; doi:10.3390/cancers16152714)
Supplement: Supplementary file 1 [file cancers-16-02714-s001.zip › Scheme S1.pdf]

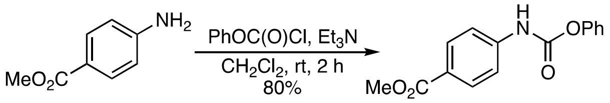

Ethyl 4-((phenoxycarbonyl)amino) benzoate. The solution of phenyl chroloformate (1.0 g, 7.0 mmol, 0.8 ml) and Et_3N (0.6 g, 6.0 mmol, 0.8 mL) in CH_2Cl_2 (25 mL) was degassed and purged with N_2. Ethyl 4-aminobenzoate (6.0 mmol, 1.00 g, 0.9 mL) was added, and the mixture was stirred at room temperature (RT) for 2 hours (hr). The reaction mixture was then diluted with H_2O (50 mL) and EtOAc (50 mL), transferred to separatory funnel, and the phases separated. The aqueous phase was extracted with EtOAc (3 x 25 mL), and the organic layer was washed with H_2O (2 x 50 mL), dried (Na_2SO_4), and concentrated under reduced pressure. The resulting crude residue was purified by flash chromatography eluting with hexanes/EtOAc (4:1) to provide the title compound (1.45 g, 80%) as a white solid. The ^{1}H NMR and ^{13}C NMR spectral data matches with the literate record.[1]

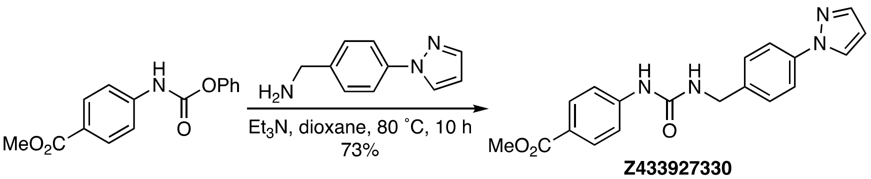

Z433927330

Z433927330. (4-(1H-Pyrazol-1-yl) phenyl) methanamine (334 mg, 1.93 mmol) and Et_3N (532 mg, 5.26 mmol, 733 µL) were added sequentially to a solution of ethyl 4-((phenoxycarbonyl)amino) benzoate (500 mg, 1.75 mmol) in dioxane (10 mL). The resulting mixture was warmed to 80 ˚C, stirred for 10 hr, then allowed to cool to RT by removal of the oil bath. The resulting crude mixture was concentrated under reduced pressure and purified by flash chromatography eluting with CH_2Cl_2/MeOH (20:1) to afford Z433927330 (475 mg, 73%) as a white solid. ^{1}H NMR (400 MHz, $CDCl_3$) δ 7.89 – 7.79 (m, 3H), 7.63 (dd, *J* = 6.3, 2.0 Hz, 1H), 7.50 (tt, *J* = 5.7, 2.4 Hz, 2H), 7.41 – 7.34 (m, 2H), 7.30 (dd, *J* = 8.5, 6.0 Hz, 2H), 6.40 (dt, *J* = 6.5, 2.2 Hz, 1H), 4.34 (d, *J* = 5.6 Hz, 2H), 4.30 – 4.20 (m, 2H), 3.59 (d, *J* = 5.7 Hz, 2H), 1.30 (tq, *J* = 7.1, 3.0 Hz, 3H). ^{13}C NMR (101 MHz, $CDCl_3$) δ 166.88, 155.73, 155.67, 144.10, 141.01, 138.97, 137.77, 130.69, 128.38, 127.42, 123.38, 119.61, 119.53, 117.36, 117.26, 107.65, 77.33, 60.76, 49.62, 49.40, 49.19, 48.98, 48.76, 48.55, 48.34, 43.10, 42.98, 14.17.

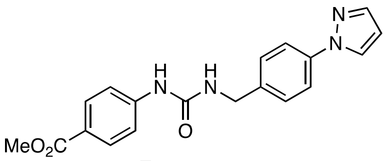
MeO2C
H
N
H
N
O
N
N

Z433927330

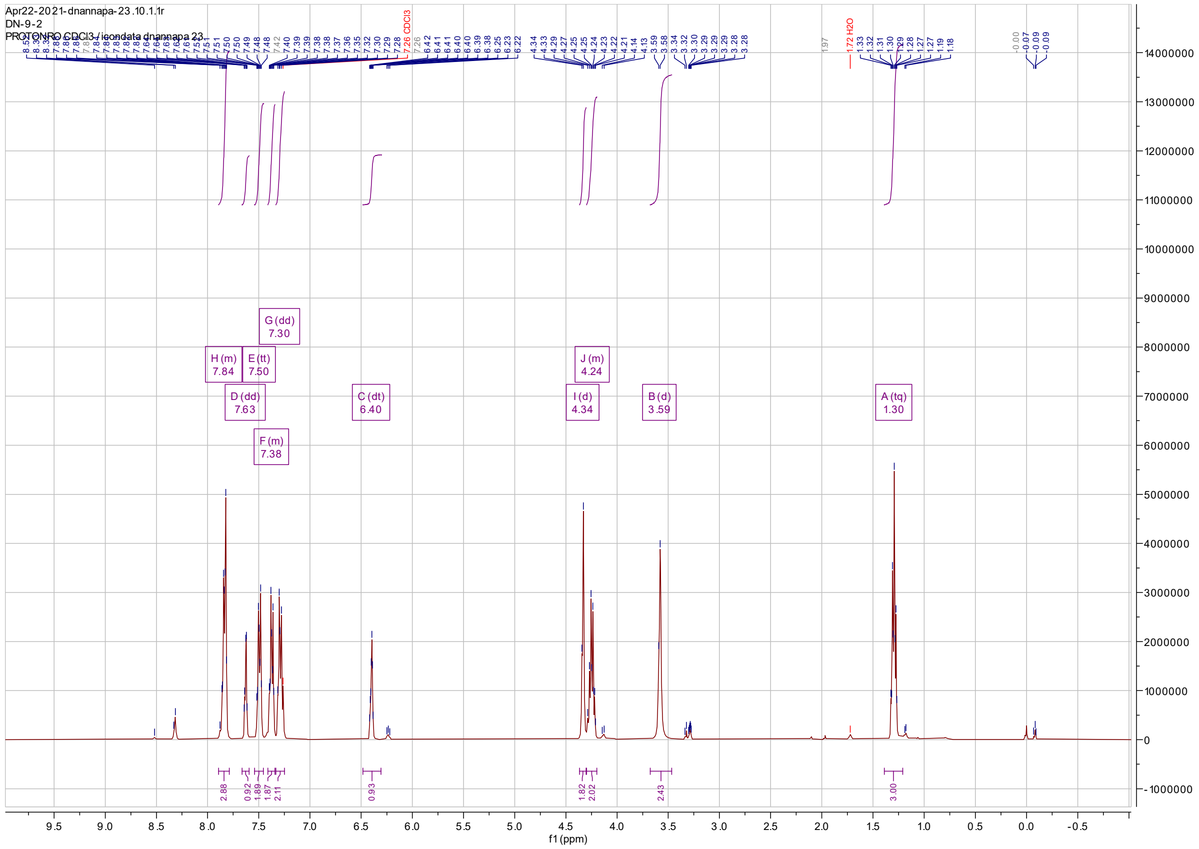
Apr22-2021-dnannapa-23.10.1.1r
DN-9-2
7.26 CDCl3
1.72 H2O
H (m)
7.84
E (tt)
7.50
G (dd)
7.30
D (dd)
7.63
F (m)
7.38
C (dt)
6.40
J (m)
4.24
I (d)
4.34
B (d)
3.59
A (tq)
1.30
2.88
0.92
1.89
1.87
2.11
0.93
1.82
2.02
2.43
3.00
f1 (ppm)

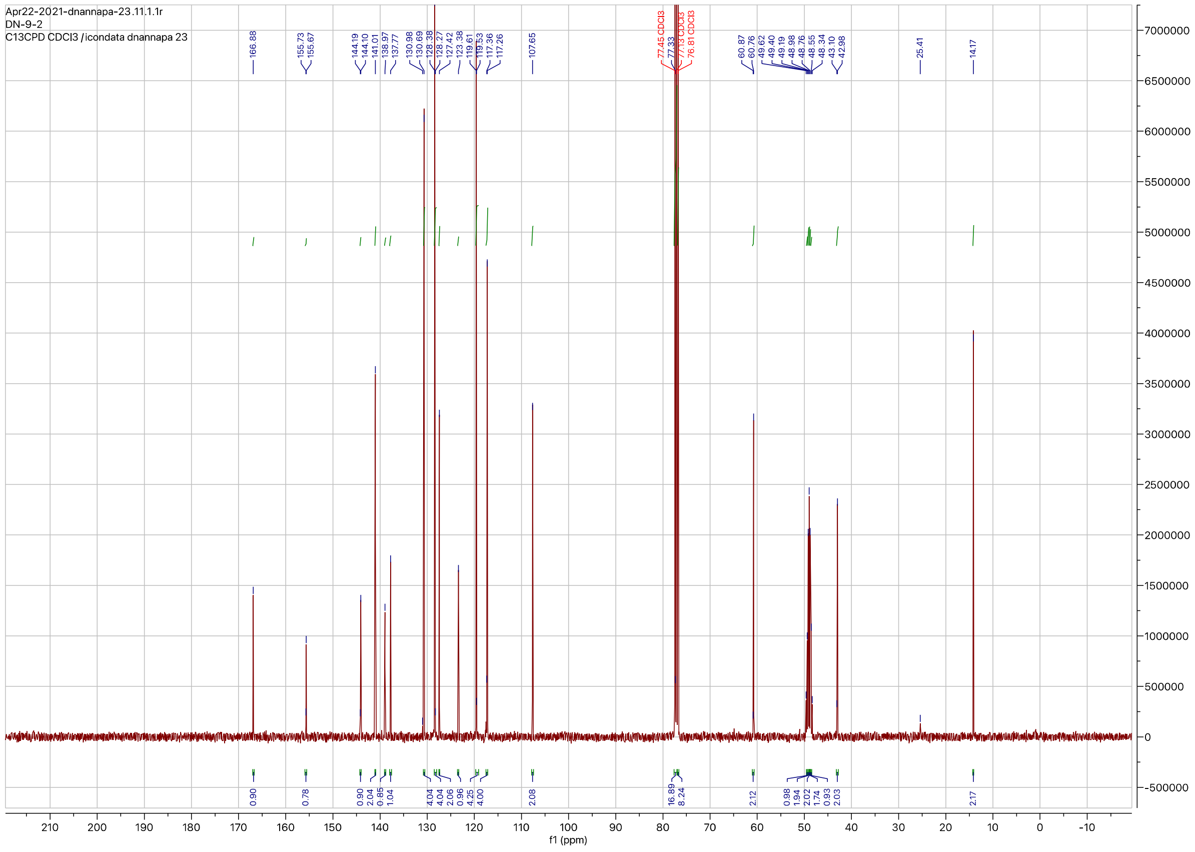

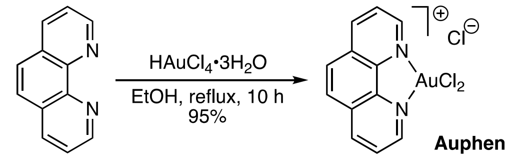

Auphen. A solution of $HAuCl_4$ (1.00 g, 3.3 mmol) in EtOH (10 mL) was added to a solution of phenanthroline monohydrate (1.18 g, 6.6 mmol) in EtOH (10 mL), heated to reflux, and then stirred for 10 h. The mixture was then cooled to rt, and the resulting yellow/orange precipitate filtered. The solid precipitate was washed with EtOH (3 x 15 mL) and then allowed to dry under ambient air to provide $[AuCl_2(phen)]$ Cl (1.41 g, 95%) as a yellow solid. ^{1}H NMR (DMSO-d_6): 9.70 (d, 2H), 9.34 (d, 2H), 8.52 (s, 2H), 8.44 (m, 2H); ^{13}C NMR (400 MHz, DMSO-d_6) δ 148.08, 142.47, 137.87, 130.07, 128.02, 126.21. The ^{1}H NMR and ^{13}C NMR spectral data obtained were consistent with those reported in literature reports.[2]
